# Efficacy of Sialendoscopy with Steroid Irrigation for Non-Lithiasic Chronic Sialadenitis: A Systematic Review and Proportional Meta-Analysis

**DOI:** 10.3390/jcm14155202

**Published:** 2025-07-23

**Authors:** Alexios Tsikopoulos, Konstantinos Garefis, Konstantinos Sidiropoulos, Stefanos Triaridis, Vasileios Nikolaidis, Iordanis Konstantinidis

**Affiliations:** 1 School of Medicine, Faculty of Health Sciences, Aristotle University of Thessaloniki, 54124 Thessaloniki, Greece; 2 2nd Academic ORL, Head and Neck Surgery Department, Aristotle University of Thessaloniki, Papageorgiou Hospital, 56403 Thessaloniki, Greece; kgarefis@hotmail.com (K.G.); vanikol@auth.gr (V.N.); jordan_orl@hotmail.com (I.K.); 3 Orthopedic Department, Eleni Dimitriou General Hospital of Florina, 53100 Florina, Greece; kcdroq@yahoo.gr; 4 School of Medicine, University of Patras, 26504 Patras, Greece; 5 1st Department of Otorhinolaryngology-Head and Neck Surgery, AHEPA University General Hospital, Aristotle University of Thessaloniki, 54636 Thessaloniki, Greece; triaridis@hotmail.com

**Keywords:** sialendoscopy, chronic sialadenitis, recurrent sialadenitis, JRP, autoimmune sialadenitis, radioactive iodine-induced sialadenitis, sine causa sialadenitis, steroid

## Abstract

**Objective:** Recent evidence has suggested that sialendoscopy should be the first-line treatment for chronic sialadenitis. This study aimed to assess the efficacy of steroid irrigation during sialendoscopy in non-lithiasic chronic sialadenitis. **Methods:** We conducted a systematic search of the databases of PubMed, Scopus, and Web of Science up to the 3rd of November 2024 for completed studies investigating the efficacy of steroid irrigation during sialendoscopy for chronic non-lithiasic sialadenitis. Primary outcomes were the number of patients with recurrence of sialadenitis symptoms and the number of patients who required a revision sialendoscopy. Secondary outcomes included the assessment of major complications of the procedure. Random-effect meta-analysis of proportion was conducted using Open Meta-Analyst software. Additionally, separate subgroup analyses linked to the underlying salivary gland disease were carried out. The quality of the included studies was evaluated utilizing the Moga tool and the ROBINS-I tool. **Results:** Thirty studies qualified for inclusion in the present systematic review. The weighted pooled proportion of recurrence after sialendoscopy by patient was 27.49% (95% CI: 21.04–34.45), whereas the revision rate was 10.64% (95% CI: 7.74–13.93). In every subgroup, the proposed intervention exhibited adequate efficacy for the symptomatic relief of patients, with similar rates of revision and repetition. The rate of major complications per patient was 0.77%. **Conclusions:** This study demonstrates that interventional sialendoscopy with intraductal steroid irrigation may yield therapeutic benefit for patients with chronic non-lithiasic sialadenitis and could be considered a safe treatment.

## 1. Introduction

The main cause of chronic sialadenitis is obstruction of the salivary duct system. There are many etiologic factors implicated in the development of this disease, including stones and strictures, mucous plugs, thick saliva sludge, infections, polyps, and foreign bodies [1,2]. Strictures usually derive from chronic inflammatory injury to the duct wall, leading to fibrosis and progressive reduction in duct diameter [3]. Regardless of the variety of reasons behind chronic sialadenitis development, the clinical appearance remains similar. Most patients with chronic sialadenitis manifest with intraductal sialolithiasis [4]. However, in 15% to 25% of salivary duct obstructions cases, no evidence of sialolithiasis is observed, and this is attributed to strictures and stenoses [5].

Non-lithiasic chronic sialadenitis represents a diagnostic challenge given the fact that the differential diagnosis is vast. To elaborate further, various factors are implicated in the development of this condition, such as infectious and inflammatory reasons, radiation damage, radioactive iodine therapy, and autoimmune diseases [2]. Furthermore, autoimmune diseases such as IgG4-related disease [6], primary and secondary Sjögren’s syndrome [7], or systemic lupus erythematosus [8] are also linked to recurrent episodes of painful gland swelling. Additionally, chronic sialadenitis may present in patients with thyroid malignancies treated with intravascular radioactive iodine [9]. Furthermore, juvenile recurrent parotitis (JRP), an inflammatory condition of unknown etiology, is also characterized by recurrent episodes of unilateral, or less commonly bilateral, parotid swelling in children and adolescents [10,11,12]. Last but not least, another culprit might be “sine causa recurrent sialadenitis,” a rare clinical entity that refers to patients with recurrent sialadenitis without clinical or radiological signs of the etiopathology of inflammation and obstruction [13].

Previously, chronic sialadenitis was considered an irreversible disease [14]; however after the refinement of endoscopic instruments and the development of sialendoscopy, it has been shown that chronic sialadenitis is more often than not curable [15]. Sialendoscopy was introduced in Europe in 1991 [16] and involves a minimally invasive technique [17,18] that allows for direct visual access to the salivary duct system for the diagnosis and treatment of obstructive causes [11,19,20]. Although sialendoscopy was initially established to diagnose and treat sialolithiasis, nowadays sialendoscopy-assisted techniques are also used for the treatment of salivary duct stenosis and inflammatory salivary disorders [11]. Sialendoscopy has become an effective, minimally invasive procedure for the diagnosis and treatment of non-lithiasic salivary gland obstruction [3,4,21,22,23] and is considered the treatment of choice for chronic sialadenitis [13,15]. During sialendoscopy, it is possible to remove debris and mucus plaques through continuous lavage of the ductal system with isotonic saline. Removal of mucus plugs by saline irrigation is also important to prevent new pathologies from developing in the future [24]. Furthermore, with the insertion of semi-rigid endoscopes, it is also possible to directly irrigate the ductal system with steroids under visual control. This technique offers an effective route to deliver medications to the gland, since the salivary gland duct is connected directly to the gland and is widely used especially in cases of chronic inflammation. The rationale behind this method derives from the fact that salivary glands affected by chronic sialadenitis are characterized by a focal periductal infiltrate consisting mainly of T lymphocytes [25]. Since steroids have anti-inflammatory effects and inhibit T-cell activation, this method could add a potential therapeutic benefit to sialendoscopy with a reduction in periductal inflammatory activity and thus the prevention of further scarring [26,27]. Additionally, the anti-inflammatory properties of steroid irrigation are particularly useful for removing accumulated proteins in the ductal system that have leaked out due to disrupted permeability [28]. Various steroid agents have been applied in the literature, with promising results thus far. However, in spite of these advances, patients with chronic sialadenitis without sialolithiasis may have persistent symptoms, probably due to irreversible damage to the ductal system, since improvement in salivary flow is only possible if saliva-producing acinar cells are present and functioning in the glandular tissue or when the parenchyma recovers [26]. The purpose of this paper is to assess the efficacy of steroid irrigation during sialendoscopy in non-lithiasic chronic sialadenitis.

## 2. Materials and Methods

This systematic review was performed according to the Preferred Reporting Items for Systematic Reviews and Meta-Analyses (PRISMA) guidelines [29].

### 2.1. Eligibility Criteria

Retrospective and prospective trials investigating the efficacy of steroid irrigation during sialendoscopy for chronic non-lithiasic sialadenitis were compiled for the purpose of the present review. Four different groups of chronic non-lithiasic sialadenitis were investigated: juvenile recurrent parotitis, radioactive iodine-induced sialadenitis (RAIS), autoimmune sialadenitis, and sine causa sialadenitis. Studies not clearly presenting the disease of the patients involved, were excluded. Additionally, studies not mentioning the type of steroid applied were excluded. Studies with orally administrated steroids were not included either. Furthermore, only studies evaluating the major outcomes of concern were considered for this review. No age restrictions were applied. Only literature in the English language was assessed. Finally, case reports, review articles, commentaries, and editorials were excluded.

### 2.2. Literature Search

Two reviewers (A.T. and K.S.) conducted a comprehensive literature search in a blinded fashion to identify published studies evaluating investigating the efficacy of corticosteroid irrigation during sialendoscopy for chronic non-lithiasic sialadenitis. The databases of PubMed, Scopus, and Web of Science were searched until 3 November 2024 utilizing the following keywords: (“chronic sialadenitis” OR “recurrent sialadenitis” OR “autoimmune sialadenitis” OR “radioactive iodine-induced sialadenitis” OR “juvenile recurrent parotitis”) AND “sialendoscopy.” In addition, the eligibility of studies from reference lists of relevant papers was assessed. Following deduplication, the titles and abstracts of the retrieved articles were screened for suitability according to the predetermined criteria, and for the remaining articles, full texts were obtained. Discrepancies between authors in the selection of articles were resolved via discussion.

### 2.3. Data Extraction

The two researchers (A.T. and K.S.) extracted data independently on the year of publication, study design, period of patients’ recruitment, the country in which the studies were conducted, patient numbers, patient demographics, follow-up time, the type and quantity of corticosteroid applied, and outcome data (primary and secondary).

### 2.4. Outcome Assessment

The primary endpoints of the present study were the number of patients with recurrence of episodes of chronic sialadenitis after sialendoscopy with steroid irrigation and the number of patients who required revision sialendoscopy. The secondary outcome was the major complications of the procedure. By definition, major complications of sialendoscopy, namely airway obstruction, avulsion of the duct, strictures, salivary fistulas and perforations, traumatic ranulas, and lingual nerve paresthesia [30], constitute adverse events that significantly change the normal postoperative course and may need further surgical procedures for their treatment [31].

### 2.5. Statistical Analysis

A single arm meta-analysis was conducted with MedCalc statistical software (version 23.0.6) [32]. The binary random-effect (DerSimonian and Laird) method was implemented [33]. Confidence intervals were set at 95%. The recurrence and repetition rate of sialendoscopy were calculated as proportions and 95% CIs for each study, and then data were pooled to derive pooled proportions and 95% CIs.

Given the fact that there are no specific tests to assess heterogeneity in proportional meta-analysis, the statistical heterogeneity of the extracted data from eligible studies was quantified using the Q-statistic, and the I^2^ statistic was used to assess its measure. Although I^2^ was initially developed in the context of comparative data, it is also commonly utilized for the estimation of heterogeneity of proportional meta-analysis [34]. The following classification was considered: 0–40% low heterogeneity, 30%–60% moderate heterogeneity, 50%–90% substantial heterogeneity, and 75%–100% considerable heterogeneity [35]. Additionally, separate subgroup analyses linked to the underlying salivary gland disease were carried out. Of note, a *p*-value less than 0.05 indicated statistical significance in the present study. The overall results of the statistical analysis are displayed in forest plots.

### 2.6. Assessment of Quality

Concerning quality appraisal, two reviewers (A.T. and K.S.) independently assessed the risk of bias within and across prospective cohort and case–control studies utilizing the ROBINS-I tool. Additionally, for the assessment of quality for case series, the Moga score [36] was implemented and a cut-off score of 13 applied for the classification of articles into high or low quality.

### 2.7. Subgroup Analysis

We conducted separate subgroup analysis for every underlying condition, i.e., autoimmune sialadenitis, JRP, RAIS, and sine causa sialadenitis, to investigate the results of our intervention individually.

### 2.8. Sensitivity Analysis

We conducted a separate sensitivity analysis, excluding studies that could potentially lead to high heterogeneity in this study. In this study, the follow-up varied from some months to years, and hence it is obvious that the recurrence rate would be higher for longer follow-up. For this purpose, we excluded studies with follow-up longer than 36 months from the analysis or with undefined length of follow-up to attain a more homogeneous sample.

### 2.9. Analysis of Publication Bias

We utilized Egger’s test to assess for the presence of small-study effects, which acts a proxy for the assessment of publication bias.

## 3. Results

### 3.1. Study Selection

**Figure 1:**
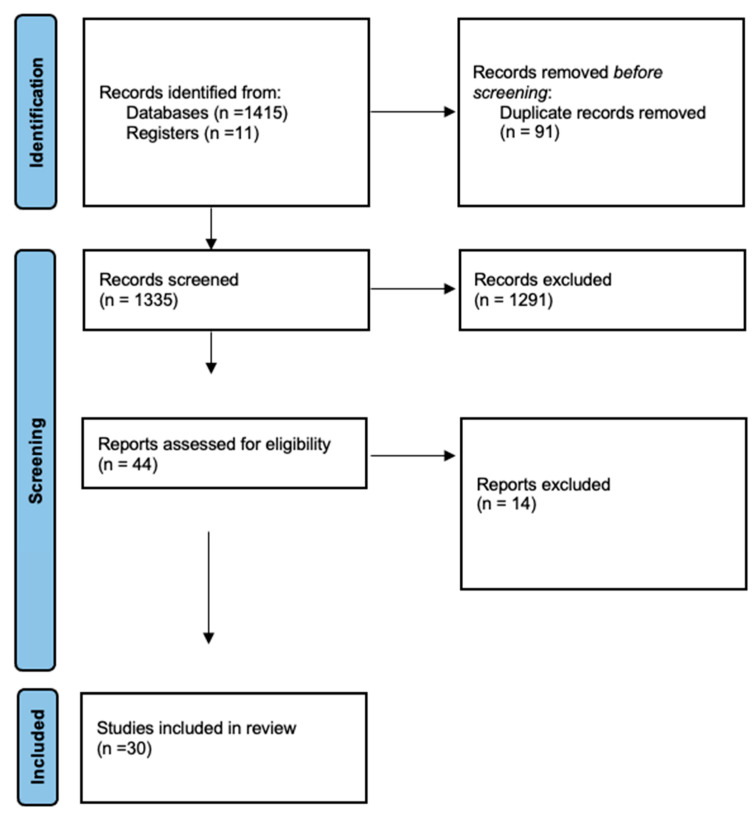
PRISMA flow diagram.

The literature search yielded a total of 1427 potentially relevant records. Following deduplication, the eligibility of the remaining 1336 studies was evaluated based on the data delivered by their title and abstracts. Ultimately, 54 articles were deemed to be relevant to the topic and were further assessed for eligibility with full-text screening. Of these, 30 studies qualified for inclusion in the present systematic review (Figure 1).

### 3.2. Study Characteristics

Overall, seven studies were conducted in the USA [15,37,38,39,40,41,42], five in Italy [13,43,44,45,46], two in Germany [47,48], two in Greece [49,50], three in India [51,52,53], one in Switzerland [54], one in Finland [55], one in Spain [56], one in France [57], one in Slovenia [58], four in Israel [8,11,59,60], one in Turkey [61], and one in Singapore [62]. The 30 synthesized studies in the present study were conducted between 2004 and 2024, and the patients included were enrolled from 1993 to 2020. Most of the studies were of retrospective design, with the exception of four [13,38,43,62].

For JRP, 156 patients were male and 108 were female. The age ranged from 1 to 21 years. The range of follow-up was 3 to 144 months. For sine causa, 34 patients were male and 50 female. The age ranged from 26 to 62, whereas the minimum follow-up was 6 months. There was no mention of maximum follow-up. When it came to RAIS, 17 patients were male and 101 female. The age ranged from 19 to 79 and the range of follow-up was 0.5 to 84 months. Finally, for autoimmune sialadenitis, 9 patients were male and 56 were female. The age ranged from 24 to 79 years, and the follow-up ranged from 0.2 to 67.7 months (Supplemental Tables S1–S8).

### 3.3. Quality Assessment

For prospective non-randomized studies, the risk of bias was low as well (Supplemental Table S9). Concerning quality appraisal of case series, a study with 14 or more “yes” responses (70%) was considered to be of acceptable quality. As such, the majority of the included papers were deemed to be at a low risk of bias (Supplemental Tables S10–S12).

### 3.4. Synthesis of Results

**Recurrence of episodes of sialadenitis**

**Figure 2:**
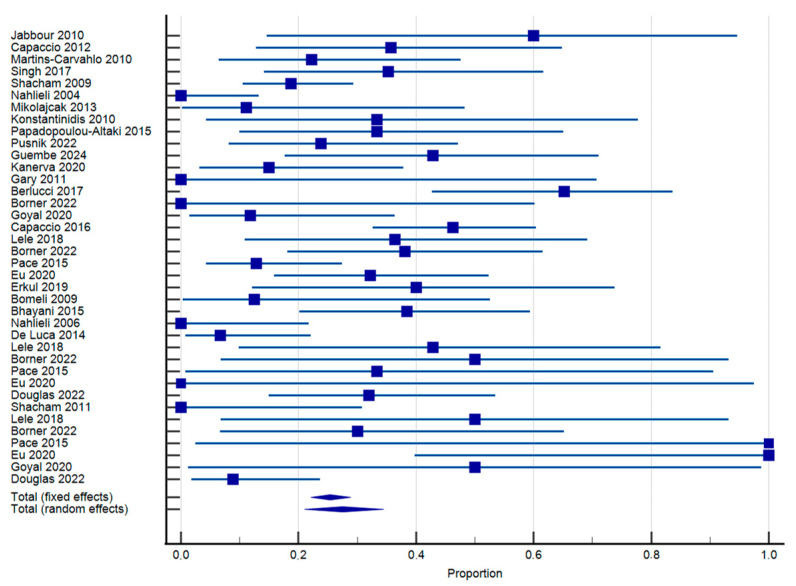
Forest plot of recurrence of episodes of sialadenitis [8,11,13,15,37,38,39,40,41,42,43,44,46,47,49,50,51,53,54,55,56,57,58,59,60,61,62].

In total, 27 studies including 631 patients suffering from chronic non-lithiasic sialadenitis qualified for the quantitative data synthesis. The weighted pooled proportion of recurrence after sialendoscopy by patient was 27.49% (95% CI: 21.04–34.45) (Figure 2). Substantial interstudy heterogeneity was found regarding this outcome (I^2^ = 70.49%; *p* < 0.0001). Likewise, subgroup analyses were implemented to assess recurrence of episodes of sialadenitis for each evaluated disease separately. For risk assessment of recurrence in the JRP group, data from 284 patients were eligible for quantitative synthesis. The weighted pooled proportion of recurrence by patient was 25.14% (95% CI: 16.12–35.41) (Supplemental Figure S1). Substantial interstudy heterogeneity was found regarding this outcome (I^2^ = 70.63%; *p* < 0.0001). Similarly, for RAIS, data from 119 patients were quantitively synthesized, and the weighted pooled proportion of recurrence was 23.15% (95% CI: 11.15–37.92) (Supplemental Figure S2). Substantial interstudy heterogeneity was found regarding this outcome as well (I^2^ = 64.96%; *p* < 0.0036). For autoimmune sialadenitis, 65 patients were included in the quantitative synthesis, and the proportion of recurrence was 39.47% (95% CI: 13.66–68.98) (Supplemental Figure S3). Again, substantial interstudy heterogeneity was detected (I^2^ = 80.32%; *p* < 0.0001). Finally, for sine causa sialadenitis, data came from 163 patients, with recurrence per patient being 33.69% (95% CI: 22–46.49) (Supplemental Figure S4). Heterogeneity was substantial (I^2^ = 62.84%; *p* = 0.0195).

**Revision sialendoscopy**

**Figure 3:**
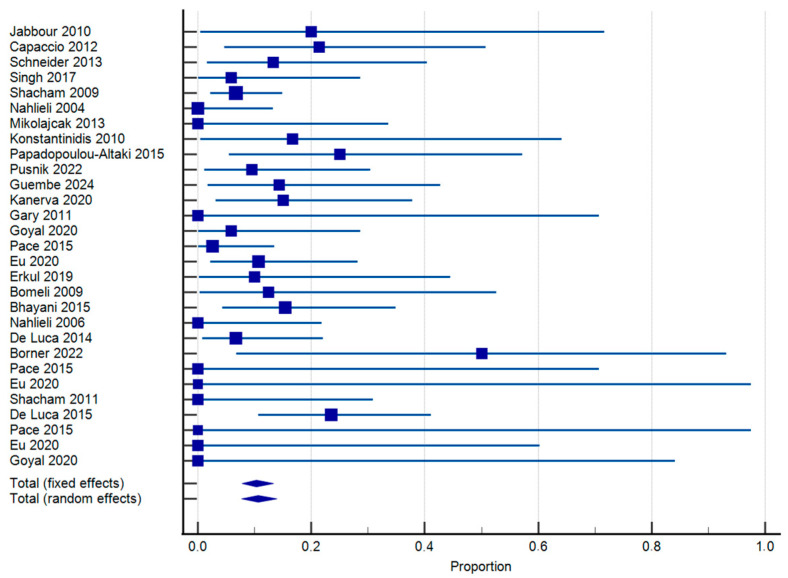
Forest plot of revision sialendoscopy [8,11,15,38,39,40,41,42,43,45,46,47,48,49,50,51,53,54,55,56,58,59,60,61,62].

Data from 24 papers including 469 patients were pooled statistically for the assessment of the need for revision of sialendoscopy per patient. As per our weighted meta-analysis findings, the revision rate was 10.64% (95% CI: 7.74–13.93) (Figure 3). Heterogeneity was low (I^2^ = 16.07%; *p* = 0.2227). For JRP, 254 were evaluated. The revision rate was 10.58% (95% CI: 6.75–15.14) (Supplemental Figure S5). Heterogeneity was found to be low as well (I^2^ = 15.85%; *p* = 0.2802). Likewise, for RAIS, 87 patients were included in the analysis. The revision rate was 11.48% (95% CI: 4.63–20.89) (Supplemental Figure S6), with low heterogeneity (I^2^ = 27.33%; *p* = 0.2199). Furthermore, for the assessment of autoimmune sialadenitis, 51 patients were included. The weighted pooled proportion of revision sialendoscopy was 12.62% (95% CI: 3.5–26.26) (Supplemental Figure S7). Heterogeneity was low (I^2^ = 23.13%; *p* = 0.2671). Finally, for sine causa sialadenitis, 77 patients were assessed, with the revision rate being 7.58% (95% CI: 2.56–14.96) (Supplemental Figure S8). Heterogeneity was again low (I^2^ = 11.02; *p* = 0.3250).

**Figure 4:**
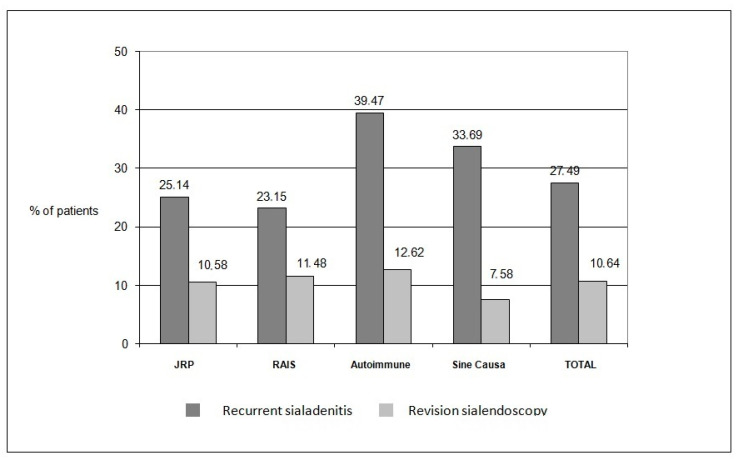
Overall rates of recurrence of symptoms and revision of sialendoscopy.

Overall rates of recurrence of symptoms and revision of sialendoscopy are depicted in Figure 4.

**Major complications**

Overall, 24 studies with 518 patients reported complications of sialendoscopy.

Major complications, i.e., adverse events that significantly alter the normal postoperative course and may require further surgical procedures, occurred in four patients. Of these, two patients suffered from ductal wall damage (0.385%) and were handled with watchful waiting, whereas the other two suffered from airway obstruction (0.385%) and an emergency tracheotomy was conducted after the procedure for the resolution of symptoms. The rest of the studies recorded no major complications. The rate of major complications per patient was 0.77%.

**Sensitivity analysis**

For episodes of sialadenitis, after statistically pooling 450 patients, the sensitivity analysis revealed a 22.87% recurrence (95% CI: 15.78–30.84). Heterogeneity was high as well (I^2^ = 70.11%; *p* < 0.0001) (Supplemental Figure S9).

For revision sialendoscopy, 322 patients were assessed. The revision rate was 10.79 (95% CI: 6.36–16.14). Heterogeneity was moderate (I^2^ = 45.95%; *p* = 0.0176) (Supplemental Figure S10).

### 3.5. Assessment of Publication Bias

For recurrence of episodes of sialadenitis, the significance level of Egger’s test was *p* = 0.2061, and thus there was no statistically significant publication bias for this outcome (Supplemental Figure S11). For revision sialendoscopy, the significance level of Egger’s test was *p* = 0.2490, and hence there was no statistically significant publication bias for this either (Supplemental Figure S12).

## 4. Discussion

Sialendoscopy has revolutionized the management of non-neoplastic salivary gland diseases and is now considered the treatment of choice for chronic non-lithiasic sialadenitis [13]. It has been postulated that the beneficial impact of sialendoscopy on these patients stems from the mechanical dilation of the ductal papilla and ductal system and removal of mucus plugs and debris with irrigation. Simple lavage of the ductal system can help remove debris and dilate the duct with hydrostatic pressure, and therefore sialendoscopy with lavage seems to be a good treatment option for patients with sialadenitis lacking sialolithiasis [63]. However, the additional intraductal administration of steroids along with sialendoscopy is commonly used in cases of chronic inflammation. Steroid irrigation can lead to local disease control and prevention of the recurrence of symptoms, adding a possible therapeutic benefit to sialendoscopy by potentially reducing the periductal inflammatory activity, increasing salivary flow, and preventing further scarring. However, the number of studies investigating the role of intraductal steroids in the management of chronic non-lithiasic sialadenitis is limited. Our study aimed to assess the effectiveness of interventional sialendoscopy with steroid irrigation for patients with recurrent non-lithiasic sialadenitis. The results of the data synthesis proved that the proposed intervention could be considered a therapeutic option to efficiently treat disease-related complaints. We found symptom resolution during the follow-up period in 72.51% of the patients with a successful procedure, whereas only 10.64% of the patients required a revision sialendoscopy for the relief of symptomatology. Still, nearly 30% of patients did not report improvement following sialendoscopy with intraductal steroid irrigation during the follow-up. This might be due to irreversible changes in the salivary gland due to chronic sialadenitis. The role of revision procedures in these patients should be further investigated [26].

Looking at the relevant literature, Plonowska et al. [64] examined prospectively patients with chronic sialadenitis without sialolithiasis. They evaluated long-term chronic sialadenitis symptoms in 27 patients following sialendoscopy along with irrigation with triamcinolone in comparison to 9 controls that were treated conservatively with antibiotic therapy alone. Seventy-two percent of cases reported partial or complete resolution of overall sialadenitis symptoms at follow-up compared to the controls (22%), *p* < 0.05. Likewise, Schneider et al. [48] compared outcomes in 15 patients subjected to sialendoscopy with steroid irrigation with those in 21 children receiving antibiotic therapy alone. However, the results of the above study showed no difference between the two groups, and the authors concluded that both therapeutic options could be considered of equal clinical merit. Regarding the additional benefit of steroid irrigation in sialendoscopy for the management of adult chronic recurrent parotitis without sialolithiasis, Jokela et al. [26] reported that single-dose steroid injection administered concurrently with sialendoscopy resulted in no improvement in VAS scores or symptom frequency at postoperative follow-up compared to saline irrigation alone. Additionally, Schwarz et al. [63] evaluated self-reported outcomes following administration of three different irrigations (normal saline, single-shot cortisone, and cortisone) for the management of sialodochitis in 94 patients. At the initial follow-up, 41 patients (43.6%) no longer had salivary gland symptoms. What is more, there were no statistically significant differences between the three intervention groups (*p* = 0.149), with the authors concluding that the type of irrigation applied during sialendoscopy could be considered of minor clinical relevance. In a study conducted by Leite et al. [65], it was demonstrated that prolonged (i.e., over 7 days) oral administration of steroids for the management of recurrent obstructive sialadenitis due to ductal stenosis significantly reduced the need for revision surgery. Despite the above findings, we highlight that high-quality RCTs are warranted to enable safe conclusions on the efficacy of steroid irrigation during sialendoscopy.

Additionally, every underlying condition leading to chronic recurrent sialadenitis has a different natural history, and the treatment responses may differ among them. Hence, given the fact that we analyzed pathophysiologically heterogeneous conditions together, i.e., autoimmune sialadenitis, JRP, RAIS, and sine causa sialadenitis, it was necessary to conduct separate subgroup analysis for every underlying condition to investigate the results of our intervention individually. Hence, we conducted subgroup analyses for patients with chronic sialadenitis depending on their underlying pathology, with the results being more than satisfactory regarding the response to the intervention. Every subgroup exhibited adequate efficacy for the symptomatic relief of patients, with similar rates of recurrence and revision, with RAIS and JRP exhibiting the best results, but not much different from autoimmune sialadenitis and sine causa sialadenitis.

Our study found a rate of symptom recurrence of 25.14% for children suffering from JRP after the proposed therapeutic intervention, and only 10.58% required a second sialendoscopy. These results verify the notion that for patients with JRP, sialendoscopy, ideally with steroid irrigation, is becoming the first-line treatment [42], reducing the number of recurrences of acute episodes of parotitis, thus offering patients a better quality of life until puberty, when the disease is usually self-limiting. The effectiveness of steroid therapy may be due to reduction in inflammation in the duct system and subsidence of the immune reactivity of periductal lymphocytes. For instance, Hackett [66] reported that 8 out of 12 patients (66.7%) with JRP were asymptomatic after sialendoscopy with steroid or steroid plus antibiotic irrigation. In a similar study by Ardekian et al. [67] with steroid and antibiotic irrigation during sialendoscopy, the rate of complete resolution of symptoms was significantly higher (86%), since there was no recurrence in 43 out of 50 patients with JRP at follow-up. Likewise, Quenin et al. [68] reported a success rate of 89% for the sialendoscopy of 17 parotid glands in 10 patients when the parotid gland was rinsed with saline solution and steroids during sialendoscopy. Only one patient needed revision sialendoscopy for recurrent symptoms. What is more, the current literature shows that sialendoscopy with steroid application is successful in treating JRP, but it is unclear whether the application of steroid alone would treat JRP equally. A retrospective study by Roby et al. [69] presented a reduction in frequency and duration of symptoms with a cure rate of 58% in 12 patients with JRP after ductal steroid irrigation through a catheter. This study shows that ductal steroid irrigation alone has similar results to sialendoscopy with steroid application, indicating that it is the steroid application and not the sialendoscopy causing improvement in symptoms. Last but not least, for all studies assessing the resolution of JRP, we must highlight that it is unknown whether the lack of recurrence within the follow-up period was the result of the natural history of the disease or of the intervention itself, and this should be taken into consideration when interpreting the results.

Another common cause of sialadenitis and strictures is radioiodine therapy. For RAIS, the proportion of recurrence was 23.15%. The revision rate was 11.48%. Still, the role of additional intervention with steroid irrigation is debated. A study using therapeutic sialendoscopy without steroid irrigation for the treatment of RAIS presented inferior results. To elaborate, Prendes et al. [70] reported complete resolution of symptoms in six patients (54%) in their study at a mean follow-up of 18 months. In contrast, Kim et al. [71] executed interventional sialendoscopy in three patients that did not respond to conservative treatment, and no symptoms recurred (100%) during a follow-up period of 8 to 10 months. Regarding treatment-resistant xerostomia after radioiodine therapy, Bulut et al. [72] conducted sialendoscopy in patients suffering from RAIS and xerostomia, and the results seemed to be beneficial when evaluating the impact on quality of life.

Our next subgroup included patients with recurrent sine causa. The postoperative recurrence rate for these patients was 33.69%. The sialendoscopy revision rate was 7.58%. This result is validated by Delagnes et al. [73] as well, who observed that the use of triamcinolone irrigation concurrently with sialendoscopy was associated with a postoperative rate of 69% for either partial or complete symptom resolution. Additionally, Vashishta et al. [20] described the therapeutic role of salivary endoscopy without steroid irrigation for idiopathic chronic sialadenitis. From a total of 51 cases, complete symptom resolution occurred in 31 patients (61%), improved with occasional symptoms in 14 patients (27%), and did not improve in 6 patients (12%) after a mean follow-up time of 20 months. The rate of recurrence (39%) was inferior to that detected by our study.

In the current study, we noticed a higher incidence of recurrence in the autoimmune sialadenitis subgroup, which included mainly patients with Sjögren syndrome (SS). Almost 40% of the patients who were subjected to therapeutic sialendoscopy along with steroid irrigation suffered from a relapse of their symptomatology. This result is quite surprising, due to the fact that the anti-inflammatory effects of steroids are expected to inhibit the focal periductal infiltrate consisting mainly of T and B lymphocytes that characterizes SS [74]. In a pilot trial, Cappacio et al. [75] sought to evaluate the role of steroid irrigation in interventional sialendoscopy in patients with sialadenitis due to SS. They included 22 patients with SS, 12 of whom were subjected to interventional sialendoscopy followed by intraductal steroid irrigations, and another 10 were subjected to interventional sialendoscopy alone. The postoperative reduction in the mean number of episodes of glandular swelling was 87% (95% CI: 77–93) and 75% (95% CI: 47–88%), respectively. Interventional sialendoscopy followed by outpatient intraductal steroid irrigations was more effective than interventional sialendoscopy alone when pain and xerostomia scores before and after treatment were analyzed together using the multivariate Hotelling T2 test (*p* = 0.0173). Regarding restoration of saliva flow, which is a major problem of SS, two studies thoroughly evaluated it. Karagozoglu et al. [76] assessed the effect of sialendoscopy on salivary flow and xerostomia in patients with SS. They randomly assigned 45 patients with SS to a control group with irrigation with saline or to a group with irrigation with saline followed by steroid application. Data regarding salivary flow were obtained up to 60 weeks after sialendoscopy. Irrespective of the irrigation protocol used, sialendoscopy resulted in increased salivary flow during follow-up up to 60 weeks. Additionally, Izumi et al. [25] also determined the efficacy of steroid irrigation of the parotid gland in relieving salivary flow deficiency in patients with SS. For this study, the parotid glands of 31 patients with primary SS were irrigated either with saline solution followed by steroid solution or with saline solution alone. They found that steroid irrigation significantly increased the salivary flow rate in patients with SS (*p* < 0.0001), with clinical improvement detectable 3.7 weeks after initial steroid irrigation.

Of note, chronic salivary gland disease might affect papilla appearance. During an episode of sialadenitis, the papilla of a salivary duct may appear elevated and the saliva produced may be thick and clumpy [77]. Various pathologies, such as chronic sialadenitis, can make the orifice of this fragile structure substantially smaller [78]. One report showed that in approximately 10% of cases, even experienced operators had problems recognizing the papilla [79].

Finally, regarding major complications, the main risk of sialendoscopy apart from ductal damage is swelling of the pharyngeal portion of the submandibular gland due to continuous saline irrigation of the duct, especially when sialendoscopy is executed bilaterally and in children, which can lead to upper-airway obstruction [80]. In our study, only two patients presented airway obstruction postoperatively and another two ductal damage.

**Strengths and Limitations**

This is the first systematic review and meta-analysis to assess the efficacy of steroid irrigation during sialendoscopy in patients with non-lithiasic chronic sialadenitis, regardless of the underlying cause. Additionally, we conducted a separate subgroup analysis that allowed for investigation of factors that potentially influence the results. What is more, the sufficient sample size in the current study allowed for a reliable investigation of the recurrence of disease and revision sialendoscopy.

However, the available evidence of the included studies must be interpreted with caution, given the fact that the included studies were mostly retrospective in nature, without control groups. Additionally, for some outcomes, substantial interstudy heterogeneity was encountered, which potentially impacts the reliability of pooled proportions, and hence no safe conclusions could be reached. Heterogeneity could be attributed to various factors. A major potential source of heterogeneity in this study could be the lack of standardized length of follow-up across studies. Follow-up varied from some months to years, and hence it is obvious that the recurrence rate would be higher for longer follow-up. We attempted to deal with this problem by conducting a sensitivity analysis excluding studies with longer follow-up than others, but heterogeneity levels remained high. Furthermore, another source of heterogeneity in the current study could be attributed to the lack of differentiation between the results of sialendoscopy in the submandibular and parotid glands because of no available data, contributing to the high heterogeneity as well. Moreover, heterogeneity is expected due to differences in the execution of the sialendoscopy. Treatment of strictures may have required the implementation of instruments such as dilators, microdrills, balloons, and endoscope tips and the insertion of stents if the stricture were localized in the ostium or the main duct in several studies. Additionally, the use of systematic steroids and antibiotics as treatment in conjunction with sialendoscopy with steroid irrigation in some of the included studies may act as a confounder and contribute to the heterogeneity of the results. Hence, the heterogeneous patient population prevents us from making strong associations between the intervention and its effect. Generally, in proportional meta-analyses, heterogeneity is usually high. This can be due to the nature of proportional data, where little variance is observed even in studies with small samples. Additionally, the current study does not provide a comparison between the administered steroid types, and we acknowledge this fact as another limitation of our study. Of note, for some subgroups the sample was small, and thus the results for the underlying conditions cannot be considered so robust. Finally, the lack of protocol registration reduces transparency and adds some risk of outcome reporting biases in this study.

**Implications for future research**

Prospective studies with a similar sialendoscopy method comparing steroid irrigation during sialendoscopy with various control groups such as saline irrigation or conservative treatment will be required to further validate the specific therapeutic value of sialendoscopy with intraductal steroid irrigation for chronic non-lithiasic sialadenitis.

## 5. Conclusions

Sialendoscopy with steroid irrigation combines the immediate symptomatic response attributed to the removal of mucus plugs and dilation of strictures with the increased therapeutic value of the localized anti-inflammatory action of steroid irrigation. We showed that evidence of moderate strength supports the use of interventional sialendoscopy with intraductal steroid irrigation for the management of chronic non-lithiasic sialadenitis, as this treatment seems to be safe and clinically effective. Nevertheless, the long-term efficacy should be investigated. We underline that RCTs are required to validate the above conclusion.

## Supplementary Materials

The following supporting information can be downloaded at https://www.mdpi.com/article/10.3390/jcm14155202/s1. Supplemental Table S1: Study characteristics for JRP, Supplemental Table S2: Study characteristics for JRP (continue), Supplemental Table S3: Study characteristics for sine causa sialadenitis, Supplemental Table S4: Study characteristics for sine causa sialadenitis (continue), Supplemental Table S5: Study characteristics for RAIS, Supplemental Table S6: Study characteristics for RAIS (continue), Supplemental Table S7: Study characteristics for autoimmune sialadenitis, Supplemental Table S8: Study characteristics for autoimmune sialadenitis (continue), Supplemental Table S9: Quality assessment for cohort studies, Supplemental Table S10: Quality assessment for case series (part 1), Supplemental Table S11: Quality assessment for case series (part 2), Supplemental Table S12: Quality assessment for case series (part 3), Supplemental Figure S1: Forest plot for recurrence of episodes of sialadenitis for JRP, Supplemental Figure S2: Forest plot for recurrence of episodes of sialadenitis for RAIS, Supplemental Figure S3: Forest plot for recurrence of episodes of sialadenitis for autoimmune sialadenitis, Supplemental Figure S4: Forest plot for recurrence of episodes of sialadenitis for sine causa sialadenitis, Supplemental Figure S5: Forest plot for revision sialendoscopy for JRP, Supplemental Figure S6: Forest plot for revision sialendoscopy for RAIS, Supplemental Figure S7: Forest plot for revision sialendoscopy for autoimmune sialadenitis, Supplemental Figure S8: Forest plot for revision sialendoscopy for sine causa sialadenitis, Supplemental Figure S9: Sensitivity analysis—Forest plot for recurrence of episodes of sialadenitis, Supplemental Figure S10: Sensitivity analysis—Forest plot for revision sialendoscopy, Supplemental Figure S11: Funnel plot of publication bias for studies regarding recurrence of episodes of sialadenitis, Supplemental Figure S12: Funnel plot of publication bias for studies regarding revision sialendoscopy.

## Registration and Protocol

The present systematic review was not registered. No protocol was prepared.
