# Supplementary material for: Efficacy of Sialendoscopy with Steroid Irrigation for Non-Lithiasic Chronic Sialadenitis: A Systematic Review and Proportional Meta-Analysis
Source: J Clin Med. 2025 Jul 23;14(15):5202. doi: 10.3390/jcm14155202 (PMC12347166; doi:10.3390/jcm14155202)
Supplement: Supplementary file 1 [file jcm-14-05202-s001.zip › Sup. Table 1 JRP.pdf]

Study (year)	Duration of Study (years)	Number of Patients	Mean Age [Range] (years)	Gender (male/female)	Steroid	Mean Follow-up [Range] (months)
Jabbour (2010)	N/A	5	6.5 [3 to 16]	5 / 0	100mg hydrocortisone	22 [7 - 33]
Cappaccio (2012)	January 2005 – January 2010	14	8 [4 to 12]	8 / 6	100mg hydrocortisone	30 [12 - 15]
Schneider (2013)	November 2004 – June 2011	15	7.5 [3 to 15]	10 / 5	100mg prednisolone	18.2
Martins – Carvahlo (2010)	January 2003 – November 2008	18	N/A	N/A	120mg prednisolone	[4 - 24]
Singh (2017)	October 2012 – September 2015	17	5.6 [3 to 11]	8 / 9	50mg hydrocortisone	[6 - 36]
Shacham (2009)	1993 - 2007	70	6 [1 to 12]	43 / 27	100mg hydrocortisone	[6 - 36]
Nahlieli (2004)	1993 - 2002	26	7 [2.5 to 13]	14 /12	100mg hydrocortisone	[4 - 36]
Mikolajcak (2013)	N/A	9	7.1 [3 to 13]	6 / 3	100mg hydrocortiosne	15 [8-26]

Konstantinidis (2010)	N/A	6	9.5 [8.3 to 11]	3 / 3	150mg prednisolone	14 [12-17]
Papadopoulou – Altaki (2015)	N/A	12	7.46 [4 to 16]	7 / 5	50 – 60mg prednisolone	25±14.4 [12 - 48]
Pusnik (2022)	September 2011 – September 2021	21	9.6 ± 5.2 [2 to 21]	18 / 3	4mg dexamethasone	48.6 [13 - 116]
Guembe (2024)	January 2010 – September 2020	14	10 [4 to 16]	N/A	4mg dexamethasone	48±36 [24 - 144]
Faizal (2017)	N/A	22	10.68 [3 to 18]	14 / 8	100mg hydrocortisone	[6 - 36]
Kanerva (2020)	September 2007 – October 2018	20	10 [3 to 16]	N/A	125mg hydrocortisone	[6 - 132]
Gary (2011)	October 2008 – November 2009	3	9 [6 to 11]	3 / 0	40mg triamcinolone acetate	9 [3 - 16]
Berlucci (2017)	June 2011 – April 2017	23	7 [4 to 12]	12 /11	100mg hydrocortisone	30 [6 - 70]

Borner (2022)	2013 - 2020	4	N/A	5 / 16	125mg methylprednisolone	minimum 6 months
Goyal (2020)	2012 - 2018	17	N/A	N/A	50mg hydrocortisone	minimum 24 months

Supplemental Table 1. Study characteristics for JRP
