# Supplementary material for: Efficacy of Sialendoscopy with Steroid Irrigation for Non-Lithiasic Chronic Sialadenitis: A Systematic Review and Proportional Meta-Analysis
Source: J Clin Med. 2025 Jul 23;14(15):5202. doi: 10.3390/jcm14155202 (PMC12347166; doi:10.3390/jcm14155202)
Supplement: Supplementary file 1 [file jcm-14-05202-s001.zip › Sup. Table 2 JRP.pdf]

| **Study (Year)** | **Post-Operative Recurrence** | **Revision Sialendoscopy** | **Major complications** |
|---|---|---|---|
| Jabbour (2010) | 3 out of 5 (first-time contralateral parotid inflammation and no ipsilateral recurrence in one patient) | 1 out of 5 | None |
| Cappaccio (2012) | 5 out of 14 | 3 out of 14 | None |
| Schneider (2013) | N/A | 2 out of 15 | N/A |
| Martins – Carvahlo (2010) | 4 out of 18 | N/A | 2 airway obstructions |
| Singh (2017) | 6 out of 17 | 1 out of 17 | None |
| Shacham (2009) | 14 out of 75 | 5 out of 75 | None |
| Nahlieli (2004) | 0 out of 26 (2 recurrences in the contralateral gland) | 0 out of 26 | None |
| Mikolajcak (2013) | 1 out of 9 | 0 out of 9 | None |
| Konstantinidis (2010) | 2 out of 6 | 1 out of 6 | None |
| Papadopoulou – Altaki (2015) | 4 out of 12 | 3 out of 12 | None |
| Pusnik (2022) | 5 out of 21 | 2 out of 21 | 2 ductal wall damage |
| Guembe (2024) | 6 out of 14 | 2 out of 14 | N/A |
| Faizal (2017) | 3 out of 22 with more than 2 episodes | N/A | N/A |
| Kanerva (2020) | 3 out of 20 | 3 out of 20 | None |
| Gary (2011) | 0 out of 3 | 0 out of 3 | None |
| Berlucci (2017) | 15 out of 23 | N/A | None |
| Borner (2022) | 0 out of 4 | N/A | None |
| Goyal (2020) | 2 out of 17 | 1 out of 17 | None |

Supplemental Table 2. Study characteristics for JRP
