# Supplementary material for: Efficacy of Sialendoscopy with Steroid Irrigation for Non-Lithiasic Chronic Sialadenitis: A Systematic Review and Proportional Meta-Analysis
Source: J Clin Med. 2025 Jul 23;14(15):5202. doi: 10.3390/jcm14155202 (PMC12347166; doi:10.3390/jcm14155202)
Supplement: Supplementary file 1 [file jcm-14-05202-s001.zip › Sup. Table 3 Sine Causa.pdf]

Study (year)	Duration of Study (years)	Number of Patients	Mean Age [Range] (years)	Gender (male/female)	Steroid	Mean Follow-up [Range] (months)
Capaccio (2016)	January 2014 – March 2015	54	53 ± 15	25 / 29	8mg betamethasone	6 months
Lele (2018)	2013 – 2016	11	N/A	N/A	40mg triamcinolone acetonide	N/A
Borner (2022)	2013 - 2020	22	N/A	5 / 16	125mg methylprednisolone	minimum 6 months
Pace (2015)	2002- 2013	39	N/A	N/A	prednisolone	6 months
Eu (2020)	January 2010 – December 2016	28	N/A	N/A	100mg hydrocortisone	N/A
Erkul (2019)	January 2011 – June 2016	10	48 [26-62]	5 / 5	8mg dexamethasone	47.1

Supplemental Table 3. Study characteristics for sine causa sialadenitis
