# Supplementary material for: Efficacy of Sialendoscopy with Steroid Irrigation for Non-Lithiasic Chronic Sialadenitis: A Systematic Review and Proportional Meta-Analysis
Source: J Clin Med. 2025 Jul 23;14(15):5202. doi: 10.3390/jcm14155202 (PMC12347166; doi:10.3390/jcm14155202)
Supplement: Supplementary file 1 [file jcm-14-05202-s001.zip › Sup. Table 4 Sine Causa.pdf]

**Study (year)**	**Post-Operative Recurrence**	**Revision Sialendoscopy**	**Major complications**
Capaccio (2016)	25 out of 54	N/A	None
Lele (2018)	4 out of 11	N/A	None
Borner (2022)	8 out of 21	N/A	None
Pace (2015)	5 out of 39	1 out of 39	N/A
Eu (2020)	9 out of 28	3 out of 28	N/A
Erkul (2019)	4 out of 10	1 out of 10	None

Supplemental Table 4. Study characteristics for sine causa sialadenitis
