# Supplementary material for: Efficacy of Sialendoscopy with Steroid Irrigation for Non-Lithiasic Chronic Sialadenitis: A Systematic Review and Proportional Meta-Analysis
Source: J Clin Med. 2025 Jul 23;14(15):5202. doi: 10.3390/jcm14155202 (PMC12347166; doi:10.3390/jcm14155202)
Supplement: Supplementary file 1 [file jcm-14-05202-s001.zip › Sup. Table 5 RAIS.pdf]

Study (Year)	Duration of Study (years)	Number of Patients	Mean Age [Range] (years)	Gender (male/female)	Steroid	Mean Follow-up [Range] (months)
Bomeli (2009)	July 2005 – April 2008	12 (8 with successful sialendoscopy)	46.5 [25-77]	0 / 12	40mg triamcinolone acetonide	[0.5 – 33]
Bhayani (2015)	March 2010 – June 2013	26 (25 with sialadenitis)	43 [19-57]	2 / 24	40mg triamcinolone acetonide	23.4 ± 12.1
Nahlieli (2006)	2002 - 2006	15	N/A	2 / 13	100mg hydrocortisone	[12 – 48]
De Luca (2014)	September 2007 - July 2013	30	52 [41-79]	6 / 24	hydrocortisone	[0.5 – 84]
Lele (2018)	2013 - 2016	7	N/A	N/A	40mg triamcinolone acetonide	N/A
Borner (2022)	2013 - 2020	4	N/A	N/A	125mg methylprednisolone	minimum 6 months
Pace (2015)	2002 - 2013	3	N/A	N/A	prednisolone	6 months
Eu (2020)	January 2010 – December 2016	1	N/A	N/A	100mg hydrocortisone	N/A
Douglas (2022)	March 2013 – May 2019	25	[24-78]	7 / 18	40mg triamcinolone acetonide	16.2 [0.7 – 48.1]

Supplemental Table 5. Study characteristics for RAIS
