# Supplementary material for: Efficacy of Sialendoscopy with Steroid Irrigation for Non-Lithiasic Chronic Sialadenitis: A Systematic Review and Proportional Meta-Analysis
Source: J Clin Med. 2025 Jul 23;14(15):5202. doi: 10.3390/jcm14155202 (PMC12347166; doi:10.3390/jcm14155202)
Supplement: Supplementary file 1 [file jcm-14-05202-s001.zip › Sup. Table 6 RAIS.pdf]

Study (Year)	Post Operative Recurrence	Revision Sialendoscopy	Major complications
Bomeli (2009)	1 out of 8	1 out of 8	None
Bhayani (2015)	10 out of 26	4 out of 26	N/A
Nahlieli (2006)	0 out of 15	0 out of 15	None
De Luca (2014)	2 out of 30	2 out of 30	None
Lele (2018)	3 out of 7	N/A	None
Borner (2022)	2 out of 4	2 out of 4	None
Pace (2015)	1 out of 3	0 out of 3	N/A
Eu (2020)	0 out of 1	0 out of 1	N/A
Douglas (2022)	8 out of 25	N/A	None

Supplemental Table 6. Study characteristics for RAIS
