# Supplementary material for: Efficacy of Sialendoscopy with Steroid Irrigation for Non-Lithiasic Chronic Sialadenitis: A Systematic Review and Proportional Meta-Analysis
Source: J Clin Med. 2025 Jul 23;14(15):5202. doi: 10.3390/jcm14155202 (PMC12347166; doi:10.3390/jcm14155202)
Supplement: Supplementary file 1 [file jcm-14-05202-s001.zip › Sup. Table 7 Autoimmune.pdf]

| Study (Year) | Duration of Study (years) | Number of Patients | Mean Age [Range] (years) | Gender (male/female) | Steroid | Mean Follow-up [Range] (months) |
|---|---|---|---|---|---|---|
| Shacham (2011) | N/A | 10 | 55.5 [31-79] | 0 / 10 | 100mg hydrocortisone | 14 [5-30] |
| Lele (2018) | 2013 - 2016 | 4 | N/A | N/A | 40mg triamcinolone acetonide | N/A |
| Borner (2022) | 2013 – 2016 | 10 | N/A | 5 / 16 | 125mg methylprednisolone | minimum 6 months |
| De Luca (2015) | September 2007 – July 2012 | 34 | 51.76 | N/A | hydrocortisone | 20.29 [5-36] |
| Pace (2015) | 2002 - 2013 | 1 | N/A | N/A | prednisolone | 6 |
| Eu (2020) | January 2010 – December 2016 | 4 | N/A | N/A | 100mg hydrocortisone | N/A |
| Goyal (2020) | 2012 - 2018 | 2 | N/A | N/A | 50mg hydrocortisone | minimum 24 months |
| Douglas (2022) | March 2013 – May 2019 | 34 | [24 – 75] | 4 / 30 | 40mg triamcinolone acetonide | 13.1 [0.2-67.7] |

Supplemental Table 7. Study characteristics for autoimmune sialadenitis
