# Supplementary material for: Efficacy of Sialendoscopy with Steroid Irrigation for Non-Lithiasic Chronic Sialadenitis: A Systematic Review and Proportional Meta-Analysis
Source: J Clin Med. 2025 Jul 23;14(15):5202. doi: 10.3390/jcm14155202 (PMC12347166; doi:10.3390/jcm14155202)
Supplement: Supplementary file 1 [file jcm-14-05202-s001.zip › Sup. Table 8 Autoimmune.pdf]

Study (Year)	Post-Operative Recurrence	Repeat Sialendoscopy	Major complications
Shacham (2011)	0 out of 10	0 out of 10	None
Lele (2018)	2 out of 4	N/A	None
Borner (2022)	3 out of 10	N/A	None
De Luca (2015)	N/A	8 out of 34	None
Pace (2015)	1 out of 1	0 out of 1	N/A
Eu (2020)	4 out of 4	0 out of 4	N/A
Goyal (2020)	1 out of 2	0 out of 2	None
Douglas (2022)	3 out of 34	N/A	None

Supplemental Table 8. Study characteristics for autoimmune sialadenitis
