# Supplementary material for: Efficacy of Sialendoscopy with Steroid Irrigation for Non-Lithiasic Chronic Sialadenitis: A Systematic Review and Proportional Meta-Analysis
Source: J Clin Med. 2025 Jul 23;14(15):5202. doi: 10.3390/jcm14155202 (PMC12347166; doi:10.3390/jcm14155202)
Supplement: Supplementary file 1 [file jcm-14-05202-s001.zip › Sup. Table 9 Quality assessment COHORT.pdf]

	Eu (2020)	Bhayani (2015)	Capaccio (2012)	Capccio (2016)
**Bias due to confounding factors**	Low risk	Low risk	Low risk	Low risk
**Bias in selection of participants into the study**	Low risk	Low risk	Low risk	Low risk
**Bias in classification of interventions**	Low risk	Low risk	Low risk	Low risk
**Bias due to deviations from intended interventions**	Low risk	Low risk	Low risk	Low risk
**Bias due to missing outcome data**	Low risk	Low risk	Low risk	Low risk
**Bias in measurement of outcomes**	Low risk	Low risk	Low risk	Low risk
**Bias in selection of reported results**	Low risk	Low risk	Low risk	Low risk
**Overall Bias**	Low risk	Low risk	Low risk	Low risk

Supplemental Table 9. Quality assessment for cohort studies
