# Supplementary material for: Efficacy of Sialendoscopy with Steroid Irrigation for Non-Lithiasic Chronic Sialadenitis: A Systematic Review and Proportional Meta-Analysis
Source: J Clin Med. 2025 Jul 23;14(15):5202. doi: 10.3390/jcm14155202 (PMC12347166; doi:10.3390/jcm14155202)
Supplement: Supplementary file 1 [file jcm-14-05202-s001.zip › Sup. Table 10 Moga Score 1.pdf]

	Jab bou r (201 0)	Sch neid er (201 3)	Martins - Carvahl o (2010)	Singh (2017)	Shac ham (2009)	Nahli eli (2004)	Mikolaj cak (2013)	Konsta ntinidis (2011)	Papa dopo ulou - Alata ki (2015)	Pusni k (2022)
**Is the hypothesis/ aim/objecti ve of the study stated clearly in the abstract, introductio n, or methods section?**	YES	YES	YES	YES	YES	YES	YES	YES	YES	YES
**Are the characterist ics of the participants included in the study described?**	YES	YES	YES	YES	YES	YES	YES	YES	YES	YES

**Were the cases collected in more than one centre?**	NO	NO	NO	NO	NO	NO	NO	NO	NO	NO
**Are the eligibility criteria (inclusion and exclusion criteria) explicit and appropriate ?**	YES	YES	YES	YES	YES	YES	NO	YES	YES	YES
**Were patients recruited consecutive ly?**	YES	YES	YES	YES	YES	YES	YES	YES	YES	YES
**Did patients enter the study at a similar point in the disease?**	NO	NO	NO	NO	NO	NO	NO	NO	NO	NO
**Did the authors**	YES	YES	YES	YES	YES	YES	YES	YES	YES	YES

**describe the intervention?**										
**In addition to intervention, did the patients receive any co-interventions?**	YES	YES	YES	YES	YES	YES	YES	YES	YES	YES
**Was loss to follow-up reported?**	NO	YES	NO	NO	NO	NO	NO	NO	NO	NO
**Are outcomes (primary, secondary) clearly defined in the introduction or methodology section?**	YES	NO	YES	NO	NO	NO	YES	YES	YES	YES

**Did the authors use accurate (standard, valid, reliable) objective methods to measure the outcomes?**	YES	NO	YES	YES	YES	YES	YES	YES	YES	YES
**Were outcomes assessed before and after intervention?**	YES	YES	YES	YES	YES	YES	YES	YES	YES	YES
**Was the length of follow-up clearly described/reported?**	YES	YES	NO	YES	YES	YES	YES	YES	YES	YES
**Were the statistical tests used to assess**	YES	YES	YES	YES	YES	YES	YES	YES	YES	YES

**the primary outcomes appropriate ?**										
**Does the study provide estimates of the random variability in the data for the primary outcomes (e.g. standard error, standard deviation, confidence intervals)?**	NO	YES	NO	NO	YES	NO	NO	NO	YES	YES
**Was the analysis of outcomes based on intention to treat?**	YES	YES	YES	YES	YES	YES	YES	YES	YES	YES

**Are adverse events that may be a consequence of the intervention reported?**	YES	NO	YES	YES	NO	NO	YES	YES	YES	YES
**Are the conclusions of the study supported by results?**	YES	YES	YES	YES	YES	YES	YES	YES	YES	YES
**Is there a competing interest statement about the type and source of support received for the study or about the relationship of the author(s) or other contributor**	YES	NO	YES	YES	NO	NO	YES	YES	YES	YES

**s with the manufacturer of the technology?**										

Supplemental Table 10. Quality assessment for case series studies (Part 1)
