# Supplementary material for: Efficacy of Sialendoscopy with Steroid Irrigation for Non-Lithiasic Chronic Sialadenitis: A Systematic Review and Proportional Meta-Analysis
Source: J Clin Med. 2025 Jul 23;14(15):5202. doi: 10.3390/jcm14155202 (PMC12347166; doi:10.3390/jcm14155202)
Supplement: Supplementary file 1 [file jcm-14-05202-s001.zip › Sup. Table 12 Moga Score 3.pdf]

	Erkul (2019)	Bomeli (2009)	Nahlieli (2006)	De Luca (2014)	Douglas (2022)	Shacham (2011)	De Luca (2015)
**Is the hypothesis/aim/objective of the study stated clearly in the abstract, introduction, or methods section?**	YES	YES	YES	YES	YES	YES	YES
**Are the characteristics of the participants included in the study described?**	YES	YES	YES	YES	YES	YES	YES
**Were the cases collected in more than one centre?**	NO	NO	NO	NO	NO	NO	NO
**Are the eligibility criteria (inclusion and exclusion criteria) explicit and appropriate?**	YES	YES	YES	YES	YES	YES	YES
**Were patients recruited consecutively?**	YES	YES	YES	YES	YES	YES	YES
**Did patients enter the study at a similar point in the disease?**	NO	NO	NO	NO	NO	NO	NO
**Did the authors describe the intervention?**	YES	YES	YES	YES	NO	YES	YES
**In addition to intervention, did the**	YES	YES	YES	YES	NO	NO	YES

**patients receive any co-interventions?**							
**Was loss to follow-up reported?**	YES	NO	NO	NO	NO	NO	NO
**Are outcomes (primary, secondary) clearly defined in the introduction or methodology section?**	YES	YES	YES	YES	YES	YES	YES
**Did the authors use accurate (standard, valid, reliable) objective methods to measure the outcomes?**	YES	YES	YES	YES	YES	YES	YES
**Were outcomes assessed before and after intervention?**	YES	YES	YES	YES	YES	YES	YES
**Was the length of follow-up clearly described/reported?**	YES	YES	YES	YES	YES	YES	YES
**Were the statistical tests used to assess the primary outcomes appropriate?**	YES	YES	YES	YES	YES	YES	YES
**Does the study provide estimates of the random variability in the data for the primary outcomes**	NO	NO	NO	NO	NO	NO	NO

**(e.g. standard error, standard deviation, confidence intervals)?**							
**Was the analysis of outcomes based on intention to treat?**	YES	YES	YES	YES	YES	YES	YES
**Are adverse events that may be a consequence of the intervention reported?**	YES	YES	YES	YES	YES	YES	YES
**Are the conclusions of the study supported by results?**	YES	YES	YES	YES	YES	YES	YES
**Is there a competing interest statement about the type and source of support received for the study or about the relationship of the author(s) or other contributors with the manufacturer of the technology?**	YES	YES	NO	YES	NO	NO	NO

Supplemental Table 12. Quality assessment for case series studies (Part 3)
