# Supplementary figures and images for: Efficacy of Sialendoscopy with Steroid Irrigation for Non-Lithiasic Chronic Sialadenitis: A Systematic Review and Proportional Meta-Analysis
Source: J Clin Med. 2025 Jul 23;14(15):5202. doi: 10.3390/jcm14155202 (PMC12347166; doi:10.3390/jcm14155202)

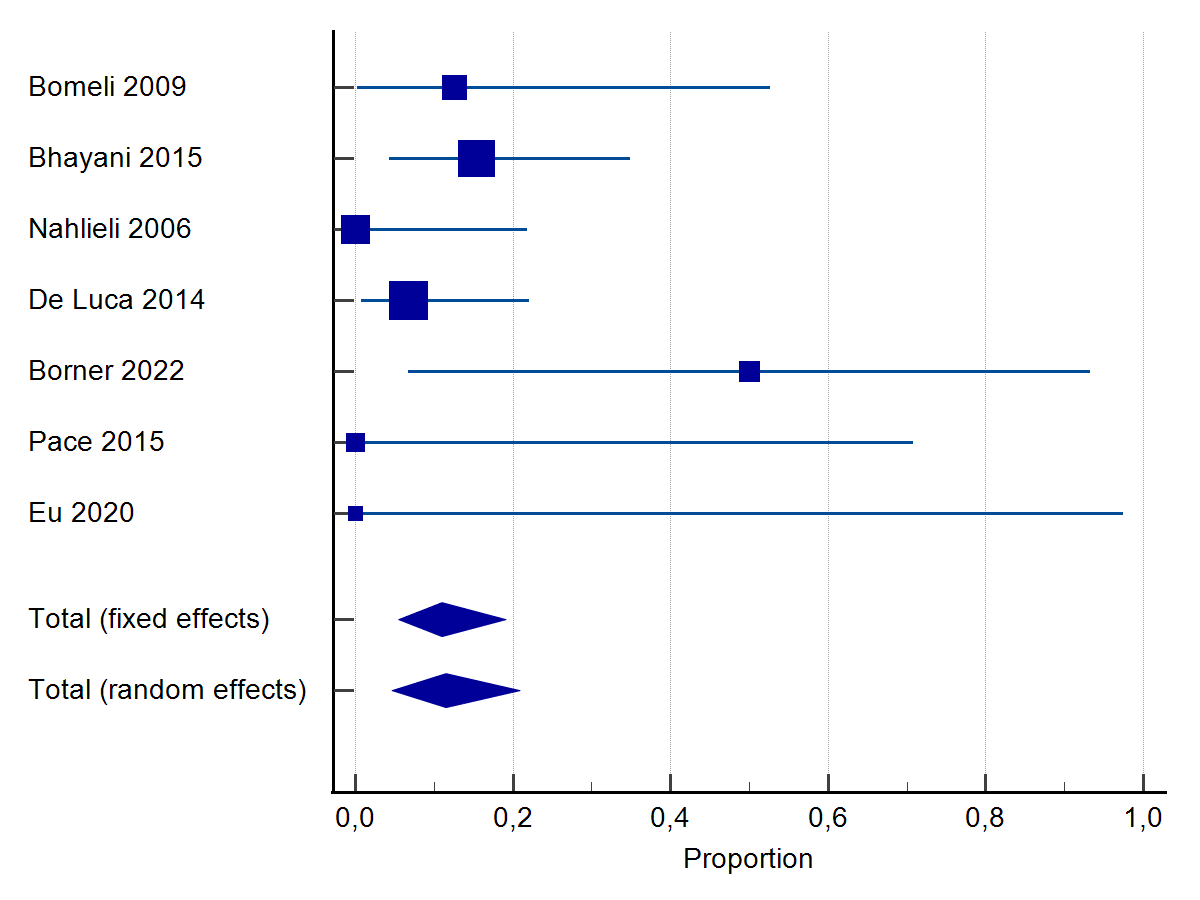

Supplement: Supplementary file 1 [file jcm-14-05202-s001.zip › Sup. Fig.6 PLOT REPEAT RAIS.png]

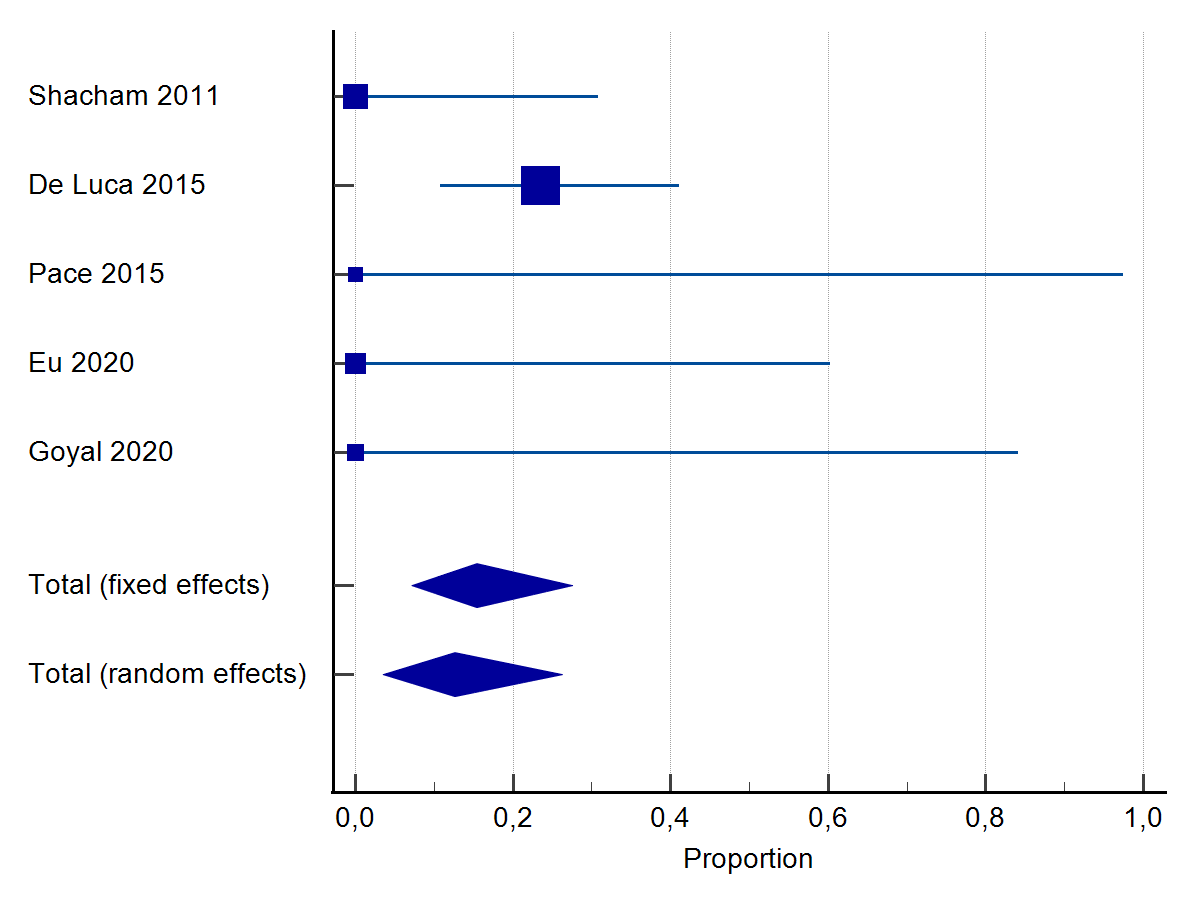

Supplement: Supplementary file 1 [file jcm-14-05202-s001.zip › Sup. Fig.7 PLOT REPEAT AUTOIMMUNE.png]

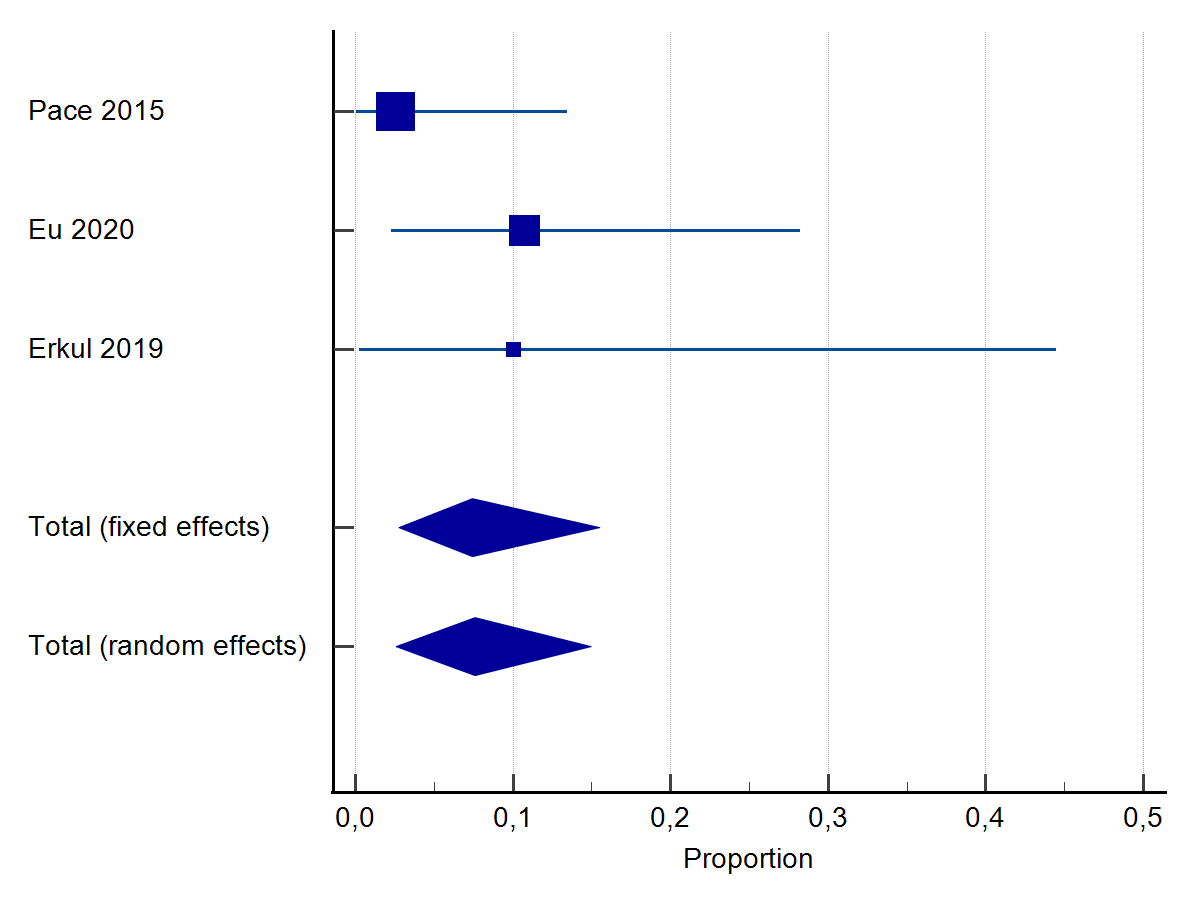

Supplement: Supplementary file 1 [file jcm-14-05202-s001.zip › Sup. Fig.8 PLOT REPEAT SINE CAUSA.png]

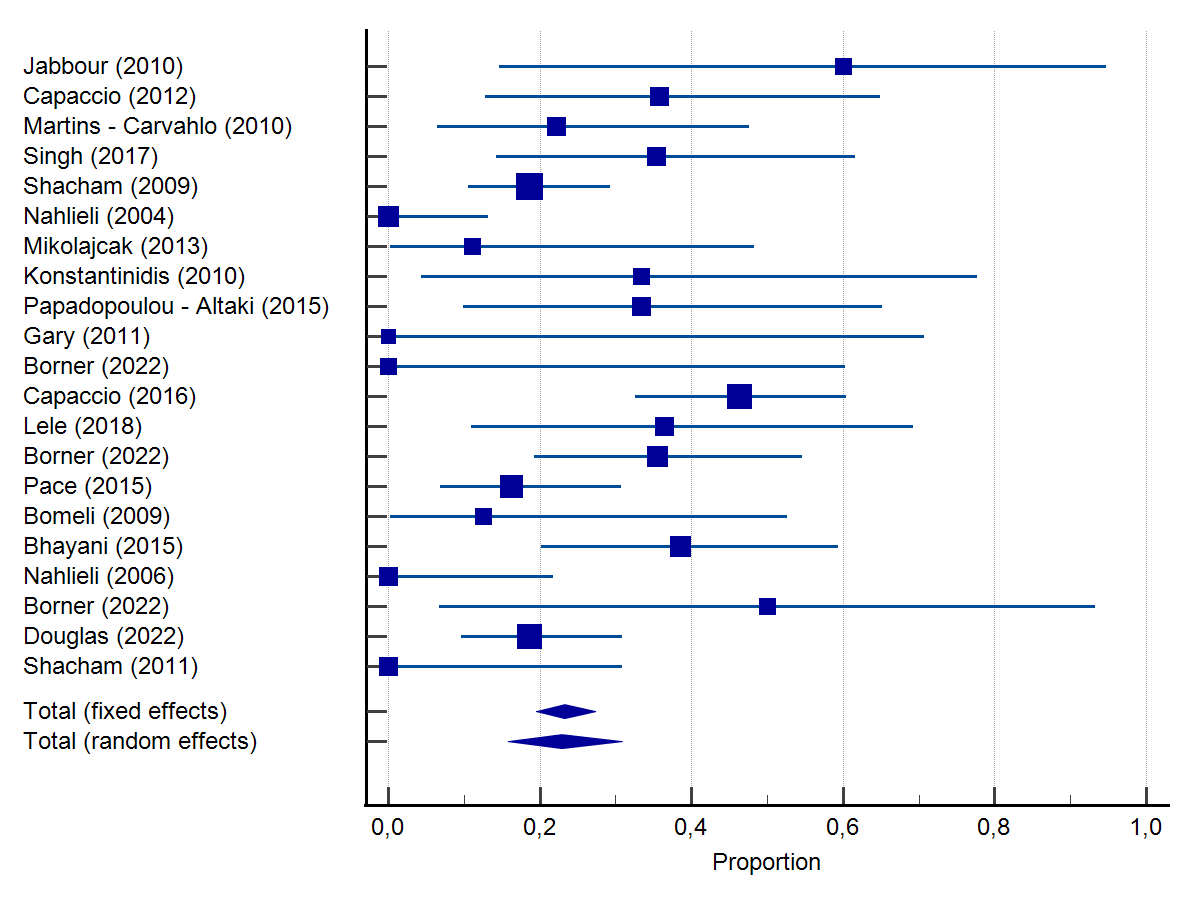

Supplement: Supplementary file 1 [file jcm-14-05202-s001.zip › Sup. Fig.9 sensitivity analysis.png]

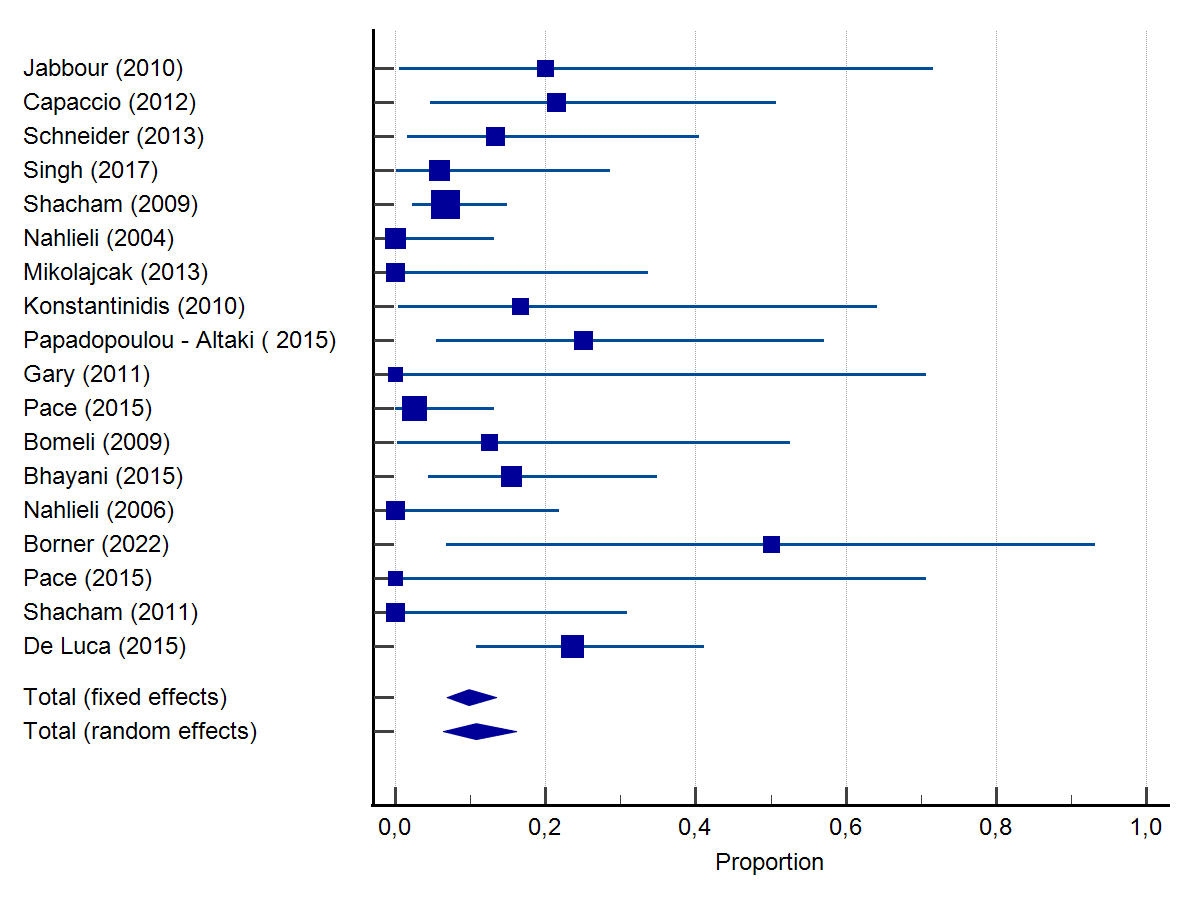

Supplement: Supplementary file 1 [file jcm-14-05202-s001.zip › Sup. Fig.10 sensitivity revision.png]

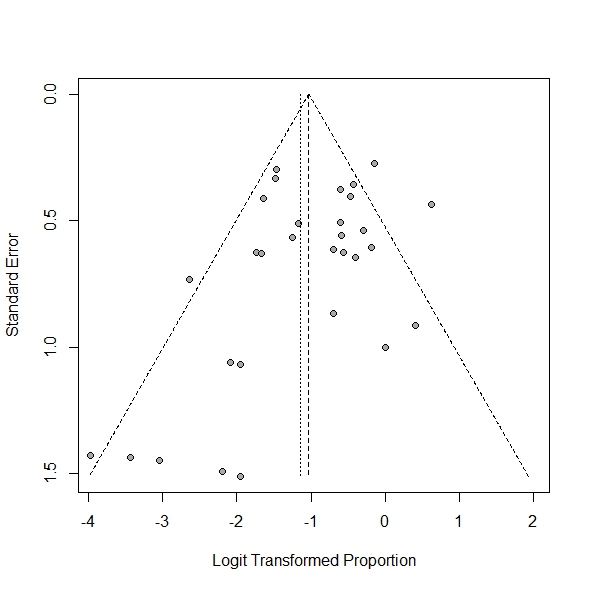

Supplement: Supplementary file 1 [file jcm-14-05202-s001.zip › Sup. FIg.11 Funnel plot 1.jpeg]

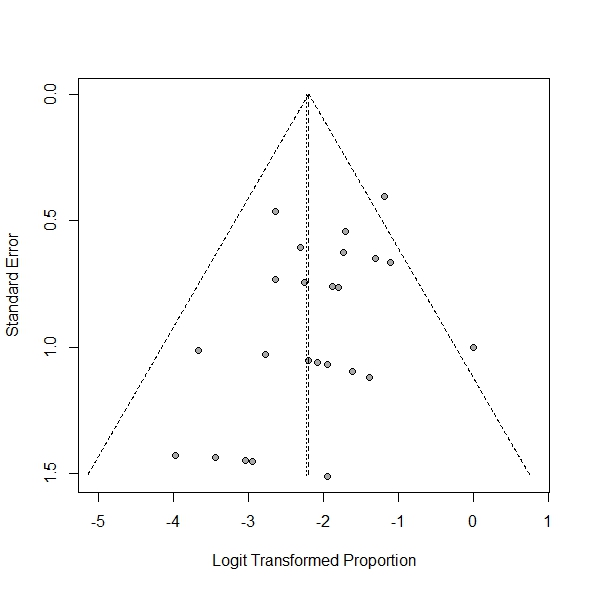

Supplement: Supplementary file 1 [file jcm-14-05202-s001.zip › Sup. Fig.12 Funnel plot 2 revision.jpeg]

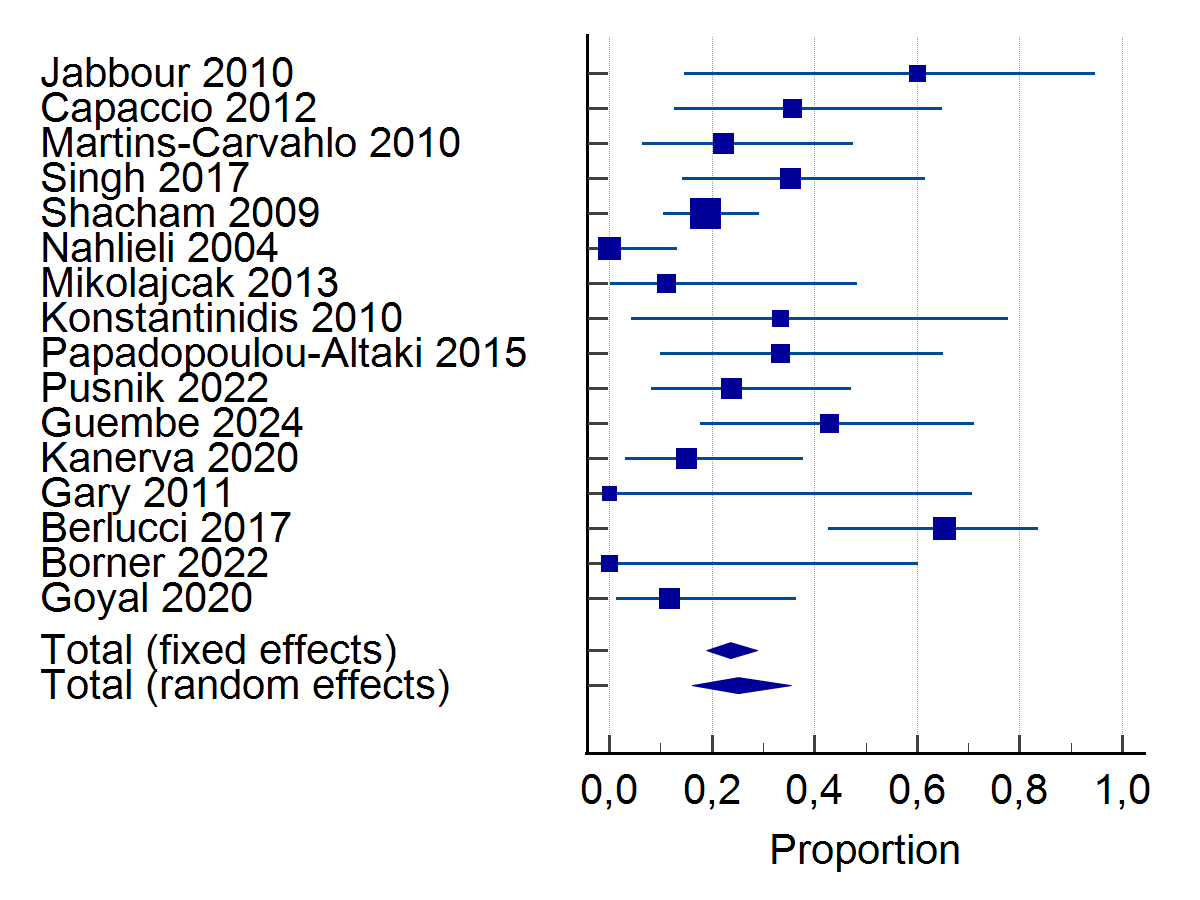

Supplement: Supplementary file 1 [file jcm-14-05202-s001.zip › Sup. Fig.1 PLOT JRP.png]

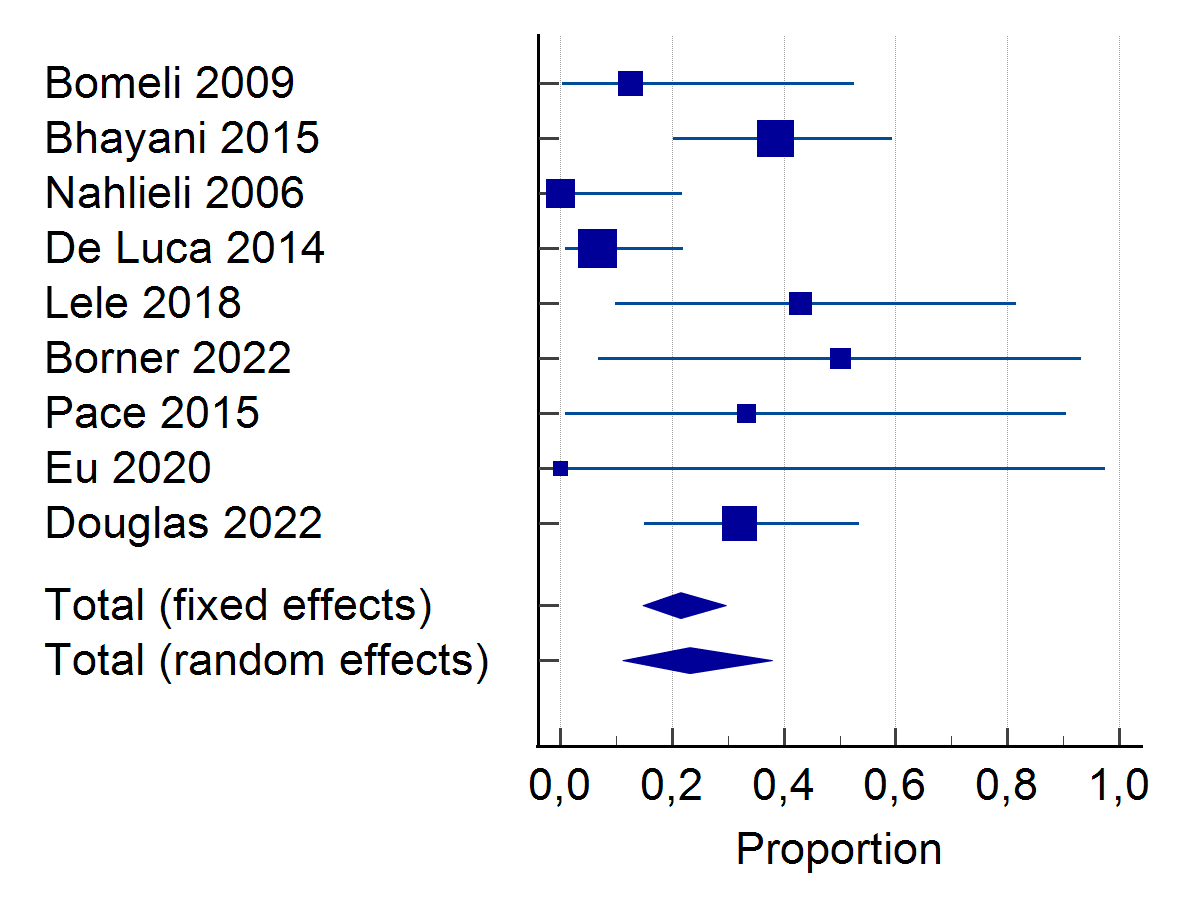

Supplement: Supplementary file 1 [file jcm-14-05202-s001.zip › Sup. Fig.2 RAIS Graph.png]

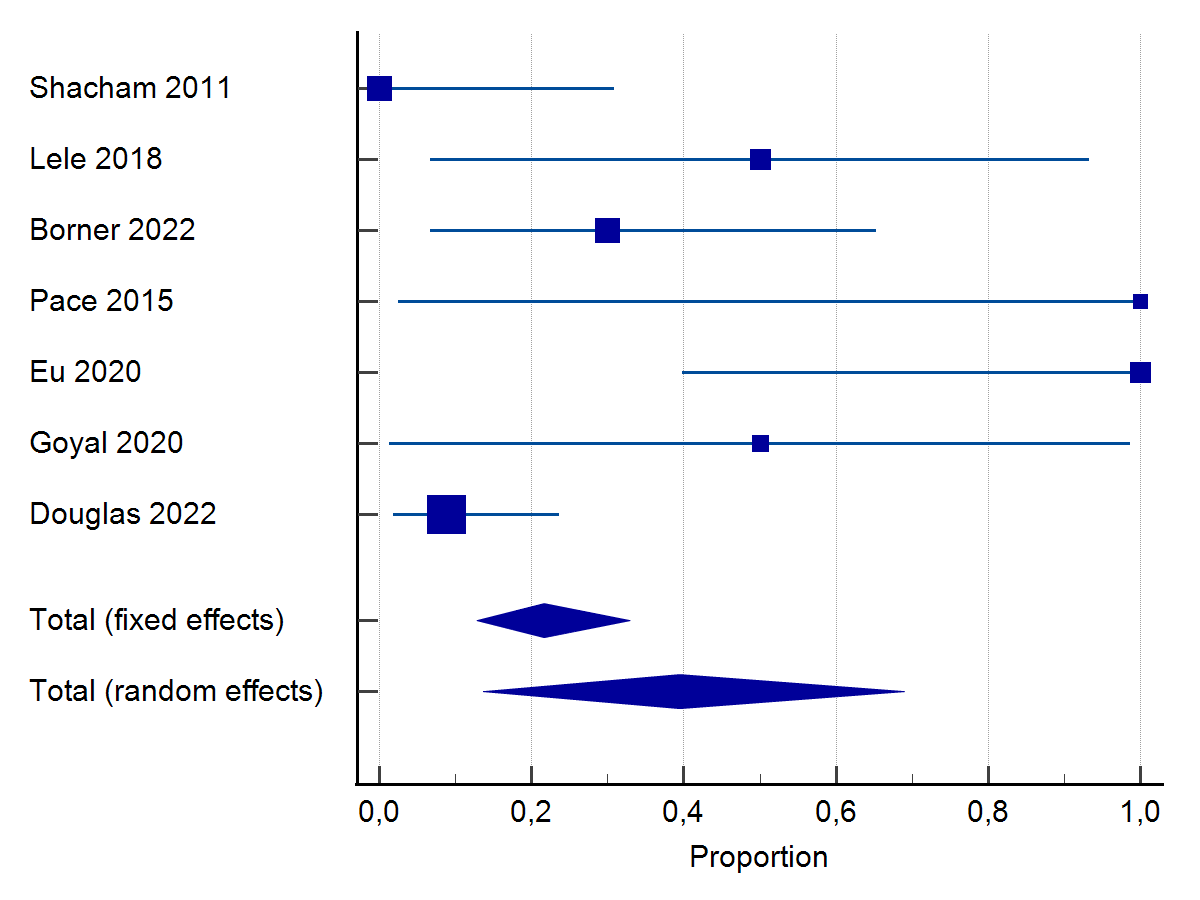

Supplement: Supplementary file 1 [file jcm-14-05202-s001.zip › Sup. Fig.3 Autoimmune Graph.png]

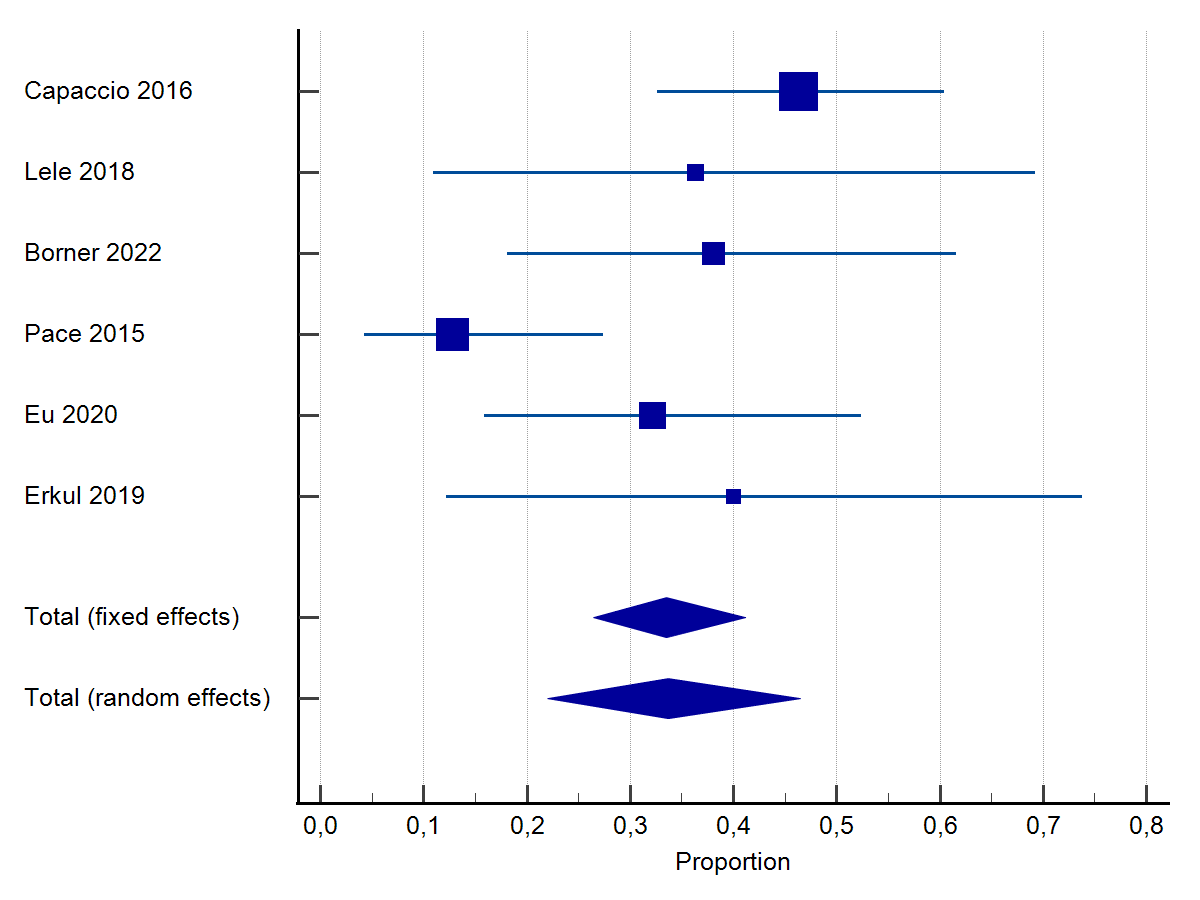

Supplement: Supplementary file 1 [file jcm-14-05202-s001.zip › Sup. Fig.4 PLOT SINE CAUSA.png]

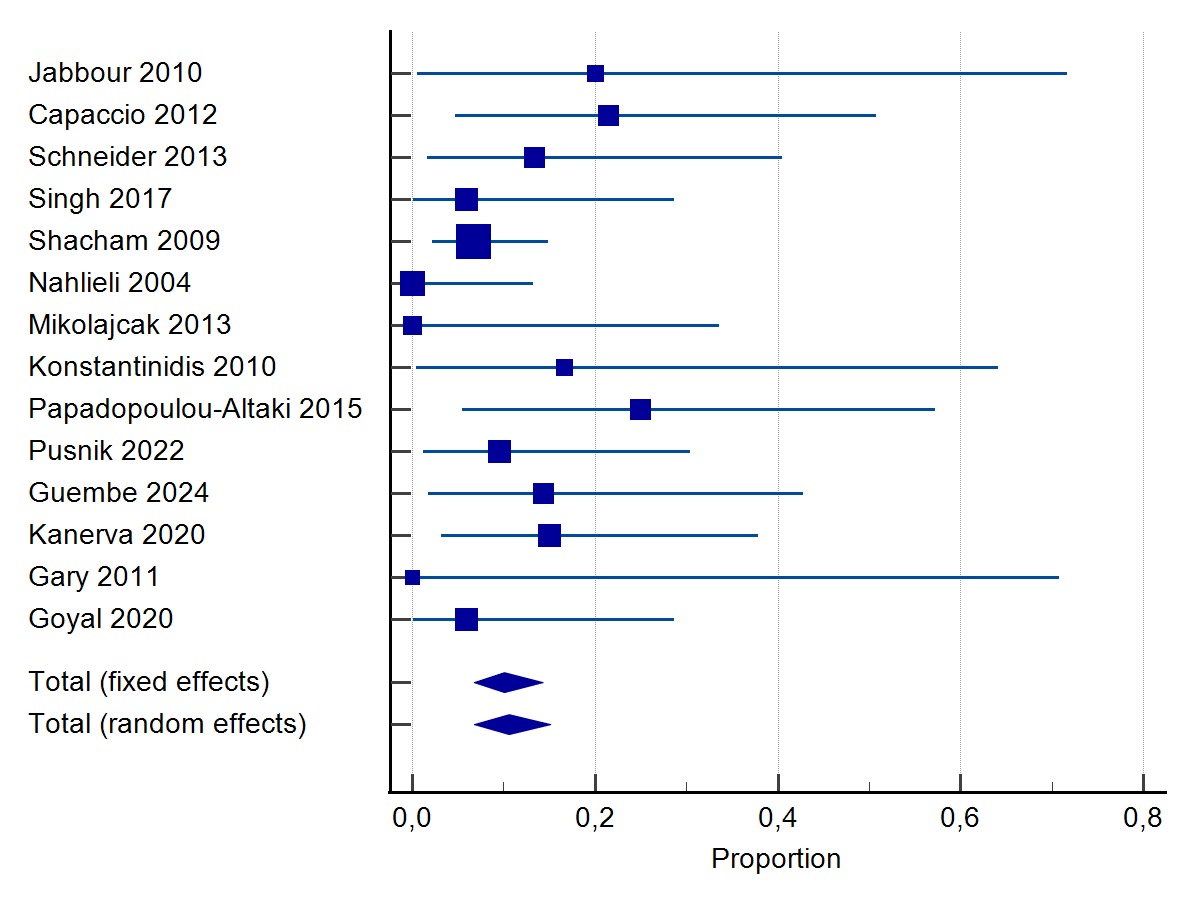

Supplement: Supplementary file 1 [file jcm-14-05202-s001.zip › Sup. Fig.5 PLOT REPEAT JRP.png]
